# Postobstructive Cyst Formation in Pancreatic Duct affecting Surgical Approach

**DOI:** 10.5005/jp-journals-10018-1272

**Published:** 2018-05-01

**Authors:** Ali Ezer, Alper Parlakgumus

**Affiliations:** 1 Department of General Surgery, Baskent University, Adana, Seyhan, Turkey

**Keywords:** Cyst, Pancreatic duct, Postobstruction, Surgical approach.

## Abstract

**How to cite this article:** Ezer A, Parlakgumus A. Postobstructive Cyst Formation in Pancreatic Duct affecting Surgical Approach. Euroasian J Hepato-Gastroenterol 2018;8(1):99-100.

Dear Editor,

Pancreatic ductal adenocarcinomas (PDACs) or pancreatic neuroendocrine tumor that may mimic a benign intraductal papillary mucinous neoplasm is the main reason of postobstructive cyst formation in pancreatic duct.^1,2^

Due to obstruction, chronical inflammation, and dilatation of the duct, these cysts may occur in some cases. Here we report a case with a postobstructive cyst formation in the pancreatic duct due to PDAC that caused a change in our surgical approach.

A 51-year-old male presented with obstructive jaundice of 1 month duration. He had deep icteric sclera with evidence of pruritus. An abdominal examination revealed mild hepatomegaly. Serum bilirubin, alkaline phosphatase, and gamma-glutamyltransferase levels were significantly elevated, while the transaminases were moderately high. A triphasic computed tomography scan revealed a well-defined lesion in the soft tissue densities in axial planes measuring 31 × 35 mm, also showing projection to the duodenal lumen localized in the head of pancreas and effecting ampulla of Vater. Due to occlusion of the pancreatic duct lesion, choledochal and intrahepatic bile duct enlargement and postobstructive cyst formation in pancreatic duct were observed (Fig. 1A). There was no invasion of major vessels. As definitive treatment, he underwent Whipple procedure. The most important circumstance in the operation was that three pancreatic ducts were encountered. One of them was the duct of Wirsung while the others were postobstructive cyst formation connected to the major pancreatic duct due to PDAC (Fig. 1B).

In our institution, our choice of anastomosis during pancreaticojejunostomy is performing end-to-side mucomucosal Wirsung-jejunostomy type. Because there were three separate structures where pancreatic juice was leaking, Wirsung-jejunostomy could not provide sufficient conditions for a secure anastomosis. As a result, we preferred a dunking pancreatic anastomosis in which the pancreas was cut 2.5 to 3 cm inside the jejunal loop. The patient was discharged without any problems on the 7th postoperative day.

Berger et al randomized 197 patients undergoing pancreaticoduodenectomy to dunking or the duct-to-mucosa technique; patients were stratified in both groups by whether the pancreatic parenchyma was hard or soft.

Pancreatic fistula occurred in 17.8% of all patients, with significantly more fistula seen in the duct-to-mucosa group compared with the invagination group (24 *vs* 12%, p < 0.05) and with more in soft glands (27%) than in hard glands (8%).

The authors concluded that the pancreatic texture was the most important factor in postoperative fistula formation.^3,4^ In addition, the diameter of the duct is the other determinant in postoperative fistula formation. The choice of the technique depending on postobstructive cyst formation seems to be another important factor when this patient is taken as example. To our knowledge this is the first published case presentation recommending dunking pancreatic anastomosis in the case of postobstructive cyst formation in pancreatic duct due to PDAC.

**Figs 1A and B:**
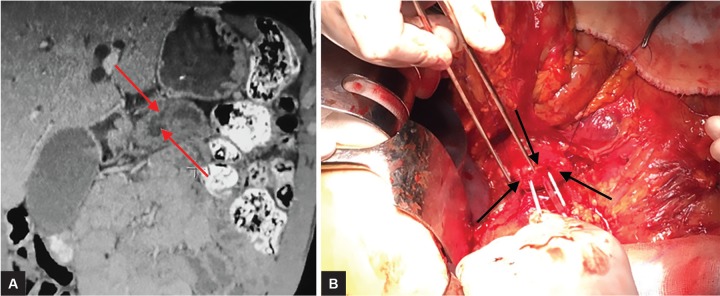
(A) Due to occlusion of the lesion dilated pancreatic duct (lower red arrow) and postobstructive cyst formation in pancreatic duct (upper red arrow) were seen. (B) Operative view of cut edge of pancreas and cannulated duct of Wirsung (upper black arrow) and postobstructive cyst formation connected to the major pancreatic duct due to PDAC (lower black arrows)
